# Editorial: Training and education in neurosurgery: strategies and challenges for the next ten years, volume II

**DOI:** 10.3389/fsurg.2024.1536176

**Published:** 2025-01-06

**Authors:** Daniele Bongetta, Cesare Zoia

**Affiliations:** ^1^ ASST Fatebenefratelli Sacco, Milan, Italy; ^2^ Ospedale Moriggia Pelascini, Gravedona ed Uniti, Italy

**Keywords:** training, education, neurosurgery, technology, simulation

**Editorial on the Research Topic**
Training and education in neurosurgery: strategies and challenges for the next ten years, volume II

What makes a great neurosurgeon? Is it talent, dedication? Is it luck or experience? Is it training and education? Probably, it's a good mix of all of the above. Interestingly, some of these features are modifiable, while others are not. When it comes to innate dexterity, personal dedication, or sheer luck (whether you believe in it or not!), there is little one can change: you either have them or you don't. Conversely, experience, training, and education always offer room for improvement. Therefore, we should focus on these three elements to analyze the modifiable features on the path to greatness in neurosurgery.

Let's begin with the first: experience. What exactly do we mean by “experience”? The dictionary defines it as “(the process of getting) knowledge or skill from doing, seeing, or feeling things” and also as “something that happens to you that affects how you feel” (1). Surgically speaking, this translates to “getting faster and better at performing a manual task” but also to “the ability to foresee and avoid potential complications.” But how do we get there? How do we become experienced? Is it merely by performing tasks ourselves an extensive number of times? No, it cannot be. Without active learning, one might repeat the same errors over and over again (albeit faster). That is where training, education (and knowledge-sharing) come into play.

Training and education are indeed the driving forces behind gaining experience, whether it involves learning to tie the first knot, reading an article, or performing a difficult case. Every step (and misstep!) moves us further along the path of experience. So, the magic formula is as simple as “experience = training×education”? Again, it cannot be. If that were the case, we probably wouldn't have made significant progress since the early days of neurosurgery, with only some exceptional performers emerging from a pool of mediocre peers. On the contrary, we believe that true progress is achieving a consistently high level of reliable performance from any given neurosurgeon.

In this context, beyond knowledge-sharing, one specific factor has driven the progress of medicine in general, elevating the performances of the majority us, normally skilled suregons: technological development. For example, Professor H. W. Cushing achieved unprecedented levels of survival and functional preservation in his time. However, advancements like electrical cautery and microscopic techniques made it significantly easier to achieve those outcomes just a few decades later. Similarly, Professor G. M. Yasargil reported that an experienced surgeon could “feel” with his instruments the difference between high-grade gliomas and the surrounding brain tissue at the interface. Nowadays, fluorophores, intraoperative imaging, and neuronavigation (such as 5-ALA or fluorescein) allow anyone to gain that insight (Sulangi et al.). Lastly, Professor C. G. Drake could effortlessly interpret the anatomy of an aneurysm from simple 2D angiographic projections. Today, a first-year resident can do so just as easily by examining a modern 3D rendering, even in a virtual or mixed-reality setting (Patel et al.). This list could go on, but the message is clear: technology repeatedly empowered surgeons with “experience” and will, hopefully, continue to do so even more over the next ten years.

What will the next game-changing advancement be, then? We believe that one of the most significant ongoing revolutions is the use of modern technology in training and education. We are finally moving beyond the traditional paradigm of books-lessons-cadavers-patients, overcoming many of its limitations through technology. For example, anatomy is now studied not only through textbooks but also via 3D renderings of normal and pathological imaging, enabling immersive experiences on smartphones (Patel et al.). Lessons are widely accessible online, and interesting surgical cases are shared daily on social media platforms. Moreover, some of the ethical and logistical constraints of cadaver training are now addressed with advanced training models that do not suffer from postmortem tissue atrophy and allow for repeated practice (de Laurentis et al., Saemann et al.). Additionally, new technologies enable progress tracking, measuring the “gain of experience” in unprecedented ways (On et al.). In this data-driven era, focused on performance metrics, transparency, and accountability, these advancements will undoubtedly be critical. Furthermore, they facilitate knowledge-sharing, a cornerstone of excellence in care. In this context, in this Reasearch Topic we present examples of how technology aids in selecting surgical approaches (Tang et al.), navigating anatomical structures (de Laurentis et al., Saemann et al.), and evaluating trainees' performances (On et al., Hanalioglu et al.). Moreover, regarding education, we showcase how technology boosts engagement and motivation among future neurosurgeons, from medical students (Unal et al.) to residents (Saemann et al.) and even fully trained surgeons. This empowerment also is proved to extend to patient education (Chatzopoulou et al.) and operating room staff (de Laurentis et al.), ensuring that everyone involved stays informed and engaged. Knowledge and experience, after all, are only valuable when shared: I learn from many, and many can learn from me. Individualism and self-referentiality must be avoided at all costs. The next ten years, with technological improvements in training devices and in inter-peer communication, will undoubtly be bound to these mantras.

In conclusion, the articles in this collection (Sulangi et al., Patel et al., de Laurentis et al., Saemann et al., On et al., Tang et al., Hanalioglu et al., Unal et al., Saemann et al., Chatzopoulou et al.) clearly show that modern neurosurgical training and education are far from merely playing with “fancy plastic toys” or indulging in technological gimmicks. They involve respectfully avoiding unnecessary cadaver or patient manipulations while enabling measurable training and enhanced knowledge-sharing.

**Figure 1:**
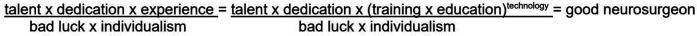
The formula for a good neurosurgeon.

Returning to our original question, in light of these considerations, we propose the following formula for a great neurosurgeon ( Figure 1 ).
